# Remarks on the Permanent Cure of Rupture; with an Account of the Different Operations Formerly Employed for That Purpose

**Published:** 1815-08

**Authors:** 


### *For the London Medical and Physical Journal.*

### *Remarks on the permanent Cure of Rupture; with an Account of the Different Operations formerly employed for that Purpose.*

AT a time when crowds of unthinking people are offering themselves as the subjects of a practice which they hear is new, and which certainly is bold, and frequently successful; it may not be an unprofitable exercise to reflect a moment, why the practice is confined to the hands of one individual, and that person so little acquainted with the principles and practice of surgery, that, if my intelligence be correct, he has offered a considerable sum annually to a regularly educated surgeon for his assistance. The explanation is not difficult. Respectable practitioners, either in medicine or surgery, have too much regard for their reputation and the welfare of their patients, to continue in the use of dangerous remedies, which, though sometimes successful, occasionally produce the most lamentable consequences. Thus we have witnessed the rise and fall of many celebrated remedies, as well as surgical operations. In the latter class I am disposed to rank the permanent cure for rupture, which has lately been revived as a modern discovery. That the effects of it are not always either pleasant or safe may be inferred from a case which was inserted in one of the Medical Journals.

An elderly gentleman in the country, sent for his surgeon to relieve him from suppression of urine. The patient stated that he was born with a rupture in his left groin; and, when he attained manhood, another rupture took place on the left side. He wore a truss, and passed, as he observed, through life fairly, until, in consequence of reading an advertisement in a newspaper, he became anxious to be blessed with a permanent cure. His own words will best explain the nature of this cure.

"They first poured some hot stuff out of a phial upon the rupture of the right side, which almost crazed me; this, they said, was to eat a hole into it. It made a large long ulcer, which they scraped clean with a silver instrument, and then they poured in some more of the stuff out of the bottle: this gave me dreadful pain. In a day or two there was an opening, into which one of them put his fore-finger, and pulled out a large piece of flesh as big as my two fingers, which he cut off with a knife. He then passed up his fingers again, and put up the rupture. The outside opening was plastered up, and soon healed. When it was nearly well, I submitted to a repetition of the treatment for the removal

 of of the rupture on the other side; but here they failed of a cure; and had it not been for the kind offices of Mr. Copeland, whom I was obliged to call in, they would certainly have killed me outright."

The account in the Journal then proceeds.

"It seems, by the description of this patient, that in the second attempt to open the cavity of the scrotum, such extensive inflammation was produced, and so much irritation propagated on to the bladder, that he did not make any water for three days; and that, although he repeatedly directed the attention of these persons to that circumstance, yet no attempt was made to relieve the distended bladder, until, through the importunity of his brother, Mr. Copeland was called in. It is unnecessary to add, that this gentleman interdicted any further application towards the cure of the rupture; he attended the patient himself afterwards, twice daily, for a considerable time, until he was able to reach his own home a few weeks back. He lingered more than a month under my care, in extreme distress from a diseased bladder, produced, no doubt, by the imprudent application of some powerful caustic to the surface of the scrotum."

In his Critical Inquiry into the Present State of Surgery, Mr. Sharp has considered the subject of permanent cure for rupture with his usual acuteness and candour. As many of your readers, and especially the advocates for permanent cure, have probably never seen his remarks, I shall beg leave to recommend them to you for insertion at the conclusion of this paper. It is time that the public mind should be informed, and that noblemen and gentlemen of distinction, who attest cures in the public prints, should become acquainted with the reasons why the practice, having once been abandoned by all respectable surgeons, is not taken up again in the present enlightened and improved age of surgery.

*London;* S. F.
*July* 10, 1815.

*Extract from the Inquiry.*—"Some of the principal means employed for this end were castration, the caustic, the punctum aureum, and the royal-stitch: the first of these methods is so cruel an operation, that it never found countenance from the learned, but was performed by itinerants* only, and even amongst them, it is said, some were ashamed to avow the extraction of the testicle, and always endeavoured to conceal it from the spectators: but, however desperate the remedy be, Dionis, its most violent adversary,† grants it was effectual; and, it is certain, if any thing can prevent the relapse of the descent of the viscera into the scro-

* Dionis, 337, 4th edit. † Ibid.

tum tum or groin, it must be the stopping up the channel through which they pass; and this is done by the ligature of the spermatic cord with its tunica vaginalis, as is practised in castration; for, when the ligature drops off, it leaves a firm cicatrix formed by a consolidation of those parts, which resists the future protrusion of the viscera.

"When the cure is attempted by a caustic, the patient uses low diet, and is kept in bed during the whole course of the treatment; both which precautions are also necessary in the other methods: when the hernia is reduced, a caustic the size of a half-crown is laid upon that part of the skin which covers the rings, and ought to be of such strength, and to lie so long, as to destroy the skin, the membrana adiposa, and the processus peritonæi, without injuring the spermatic vessels: the slough is then either to be cut out, or left to digest off, after which it is presumed, that the adhesions formed to the circumference of the rings, and to the spermatic vessels, will prove an obstruction to the descent of the viscera; but, from a great deal of experience, it has at last been discovered to be a very precarious measure; for, unless the process be destroyed as well as the fat, it will signify nothing, and it is found very difficult to ascertain the strength of the caustic to such an exactness, that it shall reach just so far without injuring the vessels themselves; so that, after a fair trial, it seems now to have fallen into general discredit.

"The punctum aureum was performed in the following manner: the patient being laid on his back, and the contents of the hernia returned into the abdomen, as is always done before any of these operations are undertaken, the surgeon makes a transverse incision through the skin and fat, down to the processus peritonæi; then, with a crooked needle, he carries a golden wire under the cord close to the rings, and, with a pair of pincers, twists the two ends of the wire so as to prevent any communication of the channel below the wire, with the channel above the wire: but it required great skill to execute this process of the operation with due exactness; for, if the stricture was made too tight, the circulation of the blood in the spermatic vessels was obstructed, and, consequently, the procreative faculty destroyed; if it was not made tight enough, the purpose of the operation was not answered. Upon these accounts it came at length into disuse, though it was at first approved of by some regular practitioners.

"*The royal suture was performed by laying bare the processus peritonæi a considerable length from the rings downwards, and then with a straight needle and waxed thread, sewing it up by the glover's stitch, in such a manner as to leave the spermatic vessels free, at the same time that the channel of the process is shut up; by which means the return of the omentum or intestine was prevented: the conceit of saving many of the king's subjects by this means, without impairing the propagating powers, gave the name of Royal Suture to the method."

* Dionis, 334.—Aquapendente, 274. Padua edit. 1666.

